# Association between the Glutathione-S-transferase T1 null genotype and esophageal cancer susceptibility: a meta-analysis involving 11,163 subjects

**DOI:** 10.18632/oncotarget.24534

**Published:** 2018-02-20

**Authors:** Feng He, Changyu Liu, Ruijie Zhang, Zhipeng Hao, Yangkai Li, Ni Zhang, Liang Zheng

**Affiliations:** ^1^ Department of Thoracic Surgery, Tongji Hospital, Tongji Medical College, Huazhong University of Science and Technology, Wuhan 430030, China; ^2^ Department of Thoracic Surgery, The Third Affiliated Hospital of Soochow University, Changzhou 213003, China

**Keywords:** Glutathione-S-transferase T1, esophageal cancer, gene polymorphism, meta-analysis

## Abstract

**Background:**

Glutathione-S-Transferase T1 (GSTT1) null genotype has been shown to be associated with the risk of esophageal cancer. However, the results remain inconsistent. Thus a comprehensive meta-analysis was conducted to assess the strength of association between GSTT1 null genotype and the risk of esophageal cancer.

**Materials and Methods:**

A literature search of PubMed, Embase, China National Knowledge Infrastructure (CNKI) and Wanfang databases up to March 31, 2017 was conducted and 30 eligible articles with 4482 cases and 6681 controls were finally recruited. The strength of correlation between GSTT1 polymorphism and the susceptibility of esophageal cancer was assessed by the crude odds ratios (ORs) with 95% confidence intervals (CIs). Subgroup analyses and sensitivity analyses were performed to further identify the association.

**Results:**

GSTT1 null genotype significantly increased the risk of esophageal cancer (OR = 1.20; 95% CI 1.04–1.40; *P* < 0.05). In a subgroup analysis by ethnicity, GSTT1 null genotype was correlated with a significantly increased risk of esophageal cancer among Asians (OR = 1.33; 95% CI 1.12–1.58; *P* < 0.05), instead of Caucasians or Africans (OR = 0.91; 95% CI 0.65–1.26; *P* > 0.05 for Caucasians and OR = 1.32; 95% CI 0.98–1.77; *P* > 0.05 for Africans). In the analysis by histological type, GSTT1 null genotype was correlated with a significantly increased risk of esophageal squamous cell carcinoma (OR = 1.34; 95% CI 1.12–1.61; *P* < 0.05), particularly among Asians (OR = 1.54; 95% CI 1.30–1.82; *P* < 0.05), but not among Caucasians or Africans (OR = 0.87; 95% CI 0.48–1.57; *P* > 0.05 for Caucasians and OR = 1.32; 95% CI 0.98–1.77; *P* > 0.05 for Africans). In addition, there is no significant correlation between GSTT1 null genotype and the risk of esophageal adenocarcinoma (OR = 0.98; 95% CI 0.71–1.35; *P* > 0.05).

**Conclusions:**

Our findings demonstrate that GSTT1 null genotype significantly increases esophageal cancer risk, particularly in Asians.

## INTRODUCTION

Esophageal cancer is the sixth leading cause of cancer-related mortality and the eighth most common cancer worldwide [1]. An estimated 455,800 new esophageal cancer cases and 400,200 deaths occurred in 2012 worldwide [2]. The two major types are esophageal squamous cell carcinoma (ESCC) and esophageal adenocarcinoma (EADC). Smoking and drinking are well-known environmental risk factors for ESCC, whereas obesity and chronic gastroesophageal refluxing are main EADC risk factors. However, only a subset of individuals exposed to those environmental risk factors develop EC, suggesting a role of host susceptibility factors. Some studies have suggested that genetic polymorphisms might explain individual differences in susceptibility to esophageal cancer [3, 4].

Glutathione-S-transferases (GSTs) are important phase II biotransformation enzymes that catalyzing the nucleophilic addition of glutathione to several hazardous xenobiotics, including phase I electrophilic and carcinogenic metabolites [5]. However, these enzymes can also activate certain chemicals that target cellular proteins and DNA to elicit detrimental carcinogenic effects through genotoxic and non-genotoxic mechanisms. One of important enzymes in GSTs family is GSTT1. GSTT1 is genetically polymorphic, and deletion polymorphism of the GSTT1 loci (null genotype) results in the loss of functional activity. Several studies have found that GSTT1 null genotype is strongly associated with susceptibility to a number of cancers, such as colorectal, renal and oral cancers *et al.* [6–8]. Previous studies have been published to estimate the association between GSTT1 null genotype and the risk of esophageal cancer, but the results are inconsistent [9–11].

To date, several meta-analysis studies have reported the association between null GSTT1 genotype and the risk of esophageal cancer. However, the results of these studies remain outdated and incomprehensive [12–14]. In the last 4 years, many case-control studies were published to estimate this association. Thus, to obtain a conclusive result about this association, we performed current meta-analysis that includes all recent publications to review and summarize the association between the GSTT1 polymorphism and the risk of esophageal cancer.

## RESULTS

### Characteristics

**Figure 1:**
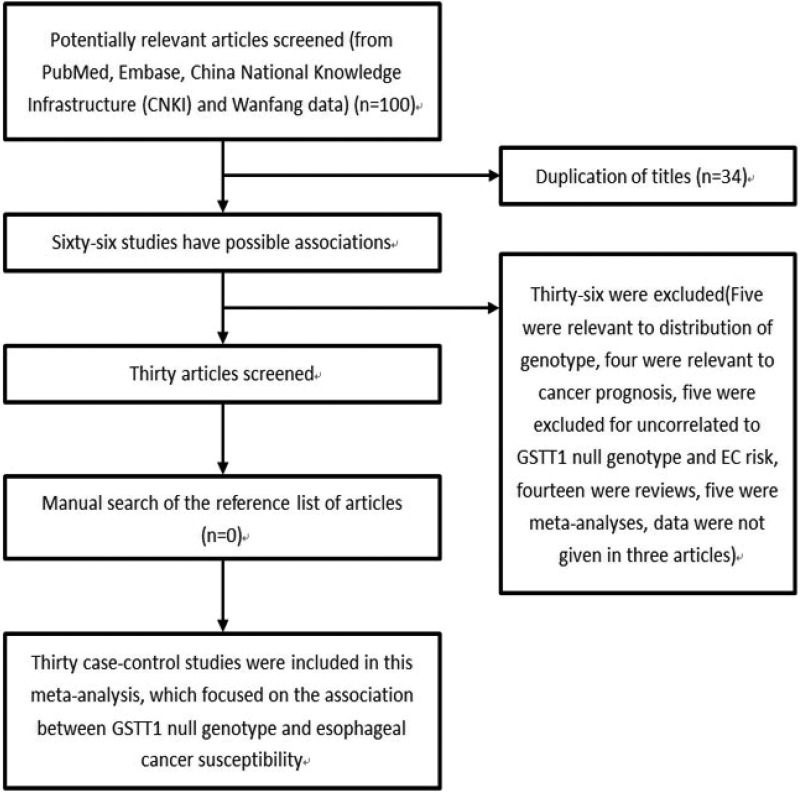
Flow chart shows studies included procedure for meta-analysis

In total, 100 articles were retrieved. Figure 1 summarized the selecting process. Finally, a total of 30 studies with 4482 cases and 6681 controls met the inclusion criteria [9–11, 15–41]. Among them, 18 were from Asians, 10 were from Caucasians, and 2 were from Africans. There were 21 studies focused on the risk of ESCC with 3272 cases and 5535 controls, and 8 studies focused on the risk of EADC with 646 cases and 1908 controls. Characteristics of included studies and the distribution of GSTT1 polymorphism are summarized in Table 1 and Table 2, respectively.

**Table 1:** Characteristics of the individual studies included in the meta-analysis

Study	Year	Country	Ethnicity	Sample size	Genotype method
Makhdoomi MA	2014	India	Asian	492/492	multiplex PCR
Sharma A	2013	India	Asian	315/436	multiplex PCR
Dura P	2013	Netherlands	Caucasian	432/591	PCR
Talukdar FR	2013	India	Asian	112/130	PCR
Zhang L	2013	China	Asian	138/170	PCR
Djansugurova LB	2013	Kazakhstan	Caucasian	107/96	PCR
Gao P	2012	China	Asian	40/80	PCR
Matejcic M	2011	South Africa	African	528/876	PCR
Malik MA	2010	India	Asian	135/195	multiplex PCR
Li D	2010	South Africa	African	238/280	PCR
Moaven O	2010	Iran	Asian	148/136	PCR
Liu R	2010	China	Asian	97/97	multiplex PCR
Ji R	2010	China	Asian	189/216	multiplex PCR
Zendehdel K	2009	Sweden	Caucasian	172/470	multiplex PCR
Zhang WL	2009	China	Asian	88/72	PCR
Deng J	2008	China	Asian	87/162	PCR
Rossini A	2007	Brazil	Caucasian	125/252	multiplex PCR
Wideroff L	2007	USA	Caucasian	67/208	PCR
Casson AG	2006	Canada	Caucasian	56/95	multiplex PCR
Jain M	2006	India	Asian	100/137	multiplex PCR
Yin LH	2005	China	Asian	106/106	PCR
Roth MJ	2004	China	Asian	131/454	PCR
Abbas A	2004	French	Caucasian	70/115	multiplex PCR
Wang LD	2003	China	Asian	62/38	multiplex PCR
Casson AG	2003	Canada	Caucasian	45/45	multiplex PCR
Ribeiro Pinto LF	2003	Brazil	Caucasian	32/67	PCR
Gao CM	2002	China	Asian	141/223	multiplex PCR
Tan W	2000	China	Asian	150/150	multiplex PCR
van Lieshout EM	1999	Netherlands	Caucasian	34/247	PCR
Lin DX	1998	China	Asian	45/45	multiplex PCR

**Table 2:** Distribution of GSTT1 null genotype among cases and controls

Study	Year	Control		EC		ESCC		EADC	
		Present	Null	Present	Null	Present	Null	Present	Null
Makhdoomi MA	2014	367	125	306	186	306	186	/	/
Sharma A	2013	373	63	233	82	233	82	/	/
Dura P	2013	463	128	335	97	87	18	248	79
Talukdar FR	2013	92	38	66	46	66	46	/	/
Zhang L	2013	90	80	62	76	62	76	/	/
Djansugurova LB	2013	35	61	19	88	19	88	/	/
Gao P	2012	55	25	18	22	18	22	/	/
Matejcic M	2011	648	228	375	153	375	153	/	/
Malik MA	2010	146	49	110	25	/	/	/	/
Li D	2010	178	102	125	113	125	113	/	/
Moaven O	2010	105	31	112	36	112	36	/	/
Liu R	2010	57	40	34	63	34	63	/	/
Ji R	2010	122	94	91	98	91	98	/	/
Zendehdel K	2009	394	76	150	22	70	7	80	15
Zhang LW	2009	39	33	31	57	31	57	/	/
Deng J	2008	75	87	36	51	/	/	/	/
Rossini A	2007	192	60	110	15	110	15	/	/
Wideroff L	2007	173	35	59	8	/	/	59	8
Casson AG	2006	80	15	42	14	/	/	42	14
Jain M	2006	100	37	72	28	56	20	6	3
Yin LH	2005	55	51	60	46	/	/	/	/
Roth MJ	2004	211	243	54	77	54	77	/	/
Abbas A	2004	85	30	56	14	31	13	25	1
Wang LD	2003	18	20	28	34	25	34	/	/
Casson AG	2003	33	12	37	8	/	/	37	8
Rebeiro	2003	52	15	26	6	/	/	/	/
Gao CM	2002	104	119	67	74	/	/	/	/
Tan W	2000	91	59	90	60	90	60	/	/
van Lieshout EM	1999	198	49	28	6	11	2	17	4
Lin DX	1998	22	23	26	19	/	/	/	/

Abbreviations: EC: esophageal cancer; ESCC: esophageal squamous cell carcinoma; EADC: esophageal adenocarcinoma.

### Quantitative synthesis

**Figure 2:**
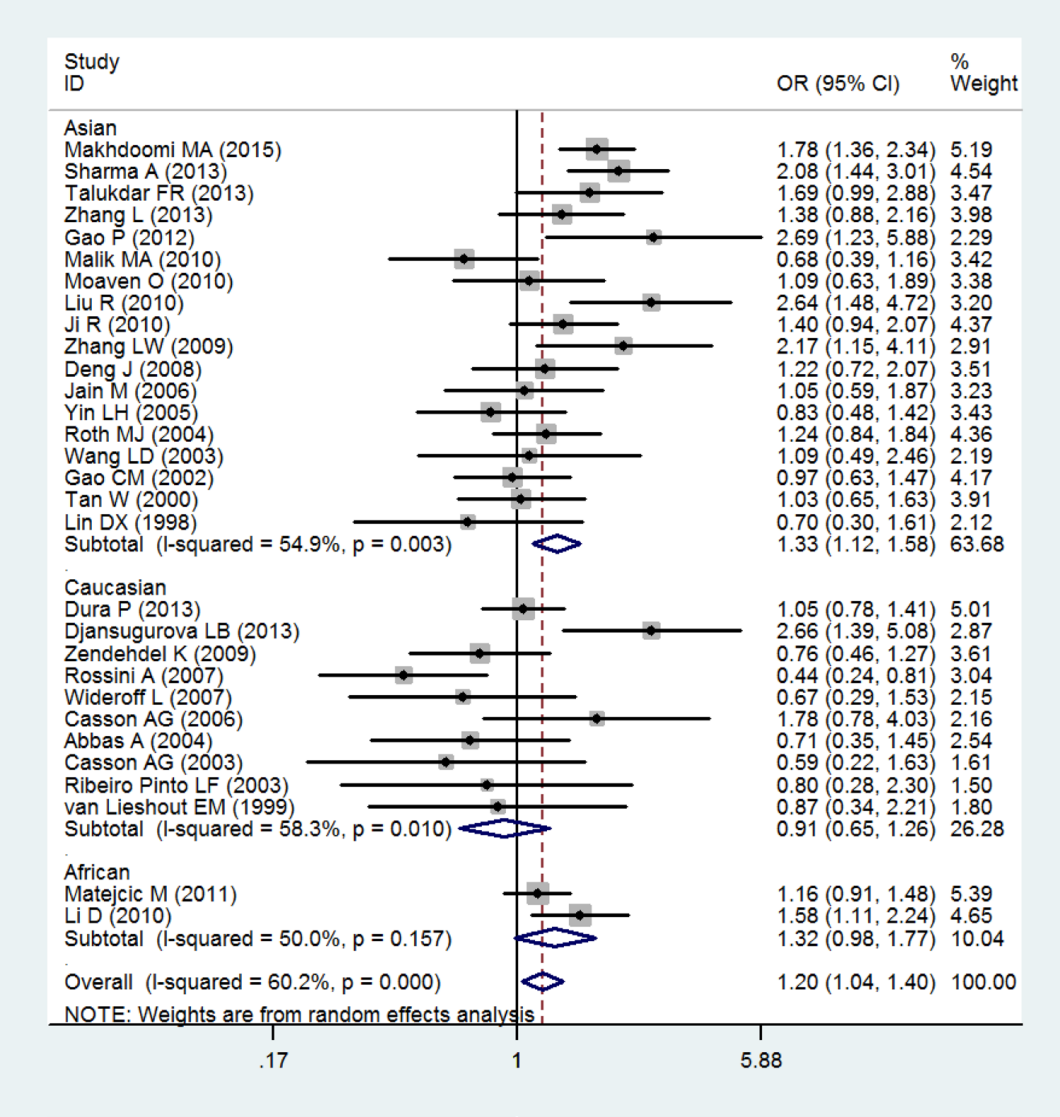
Forest plot of the association of GSTT1 null genotype with esophageal cancer risk

Table 3 showed the main result of the association between GSTT1 null genotype and the risk of esophageal cancer. Overall, there was a significant correlation of GSTT1 null genotype with esophageal cancer risk (OR = 1.20; 95% CI 1.04–1.40; *P* < 0.05; Table 3 and Figure 2). In a subgroup analysis by ethnicity, GSTT1 null genotype was correlated with a significantly increased risk of esophageal cancer among Asians (OR = 1.33; 95% CI 1.12–1.58; *P* < 0.05; Table 3 and Figure 2), but not among Caucasians and Africans (OR = 0.91; 95% CI 0.65–1.26; *P* > 0.05 for Caucasians and OR = 1.32; 95% CI 0.98–1.77; *P* > 0.05 for Africans; Table 3 and Figure 2).

**Table 3:** Meta-analysis of the GSTT1 null genotype and esophageal cancer risk

	Number of Study	OR (95%CI)	*P*	*P* (*Q*-test)	I-squared
Total	30	1.20 (1.04–1.40)	0.014	0.000	60.2
Ethnicity					
Asians	18	1.33 (1.12–1.58)	0.001	0.003	54.9
Caucasians	10	0.91 (0.65–1.26)	0.569	0.01	58.3
Africans	2	1.32 (0.98–1.77)	0.070	0.157	50.0
ESCC	21	1.34 (1.12–1.61)	0.001	0.000	62.6
Ethnicity					
Asians	13	1.54 (1.30–1.82)	0.000	0.097	35.7
Caucasians	6	0.87 (0.48–1.57)	0.640	0.002	73.5
Africans	2	1.32 (0.98–1.77)	0.070	0.157	50.0
EADC	8	0.98 (0.71–1.35)	0.911	0.240	23.7

**Figure 3:**
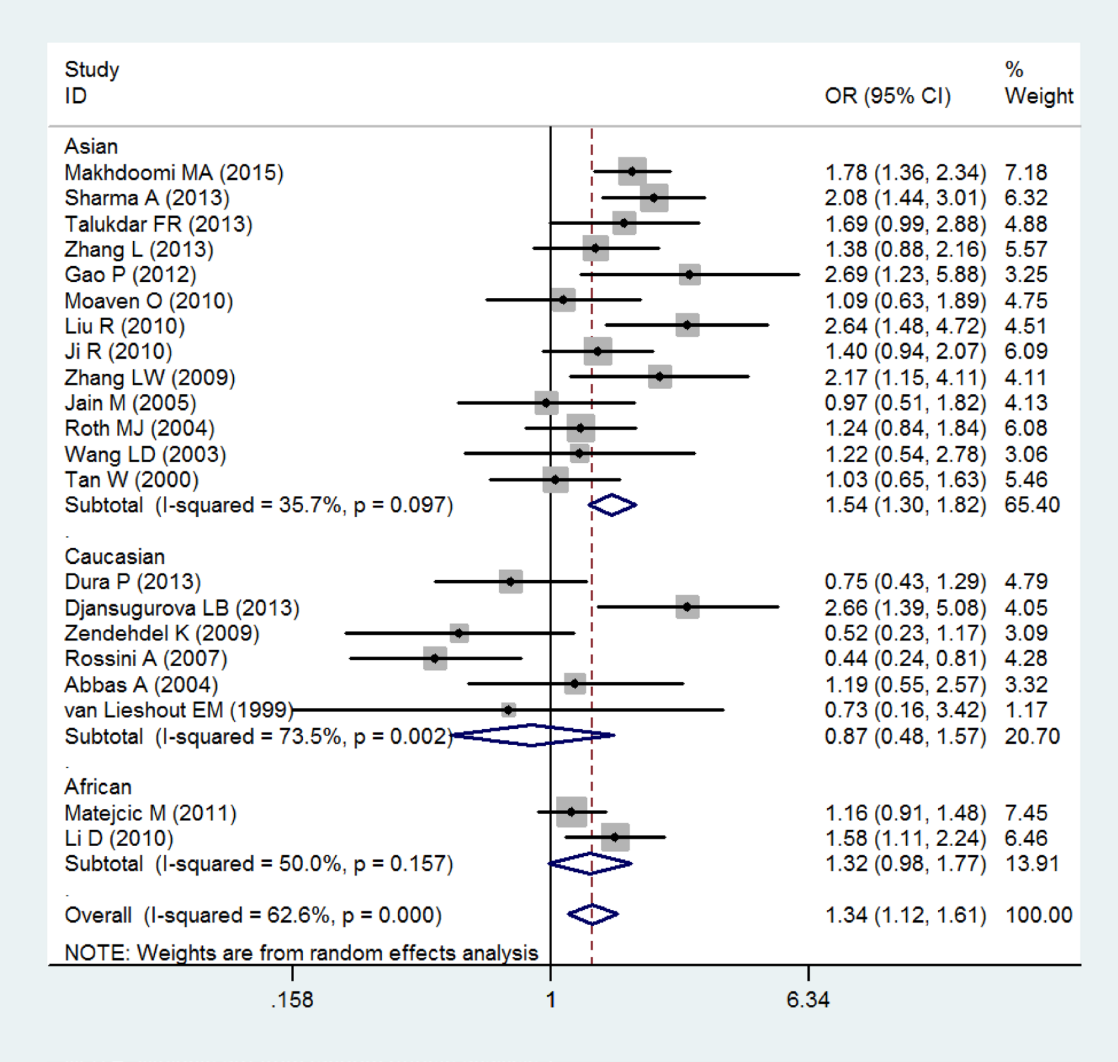
Forest plot of the association of GSTT1 null genotype with esophageal squamous cell carcinoma risk

**Figure 4:**
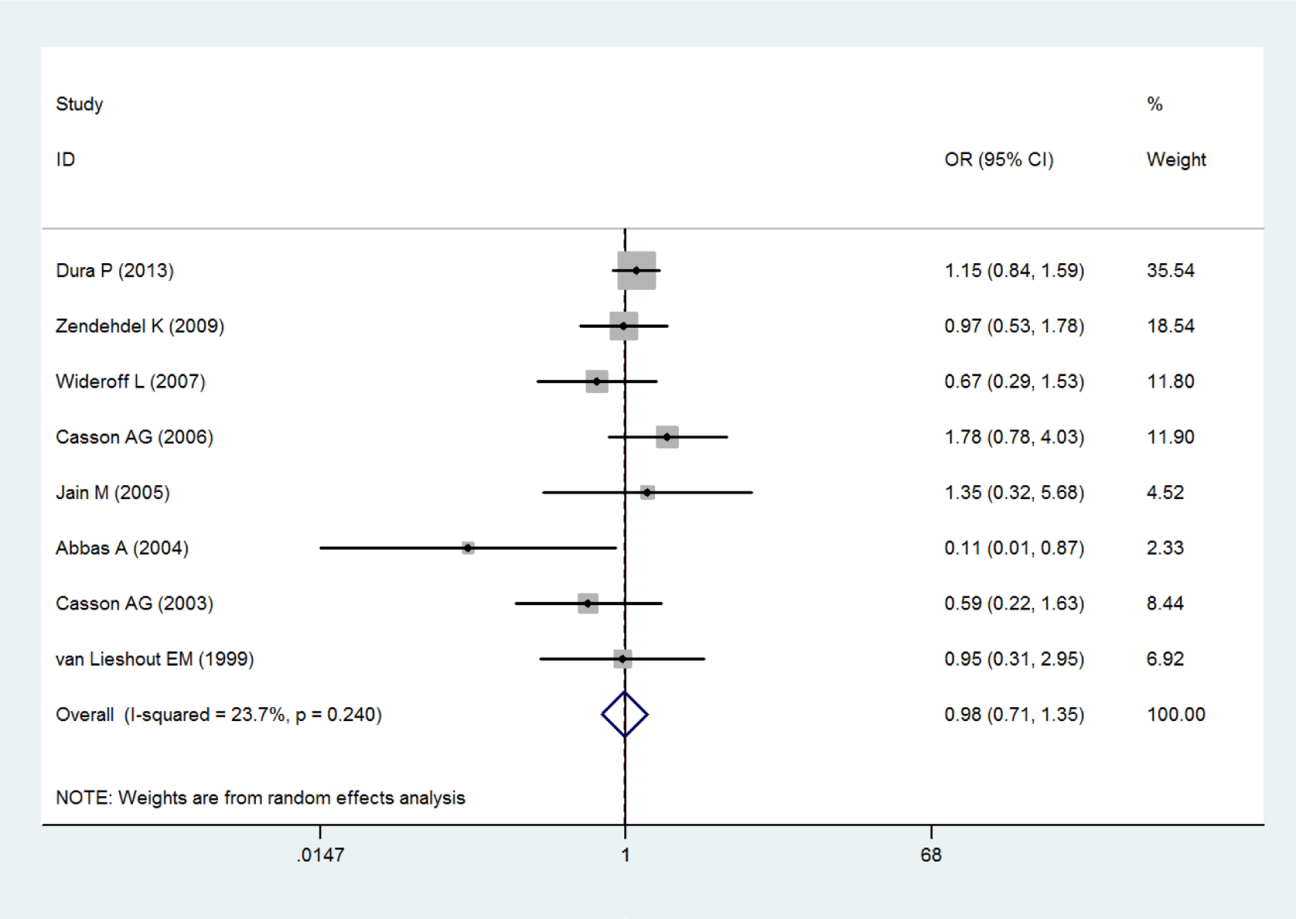
Forest plot of the association of GSTT1 null genotype with esophageal adenocarcinoma risk

In the analysis by histological type, GSTT1 null genotype were correlated with a significantly increased risk of ESCC (OR = 1.34; 95% CI 1.12–1.61; *P* < 0.05; Table 3 and Figure 3). Moreover, in a subgroup analysis by ethnicity, GSTT1 null genotype was correlated with a significantly increased risk of ESCC among Asians (OR = 1.54; 95% CI 1.30–1.82; *P* < 0.05; Table 3 and Figure 3), but not among Caucasians and Africans (OR = 0.87; 95% CI 0.48–1.57; *P* > 0.05 for Caucasians and OR = 1.32; 95% CI 0.98–1.77; *P* > 0.05 for Africans; Table 3 and Figure 3). In addition, there is no significant correlation of GSTT1 null genotype with the risk of EADC (OR = 0.98; 95% CI 0.71–1.35; *P* > 0.05; Table 3 and Figure 4).

### Test for publication bias, sensitivity analyses, and heterogeneity

**Figure 5:**
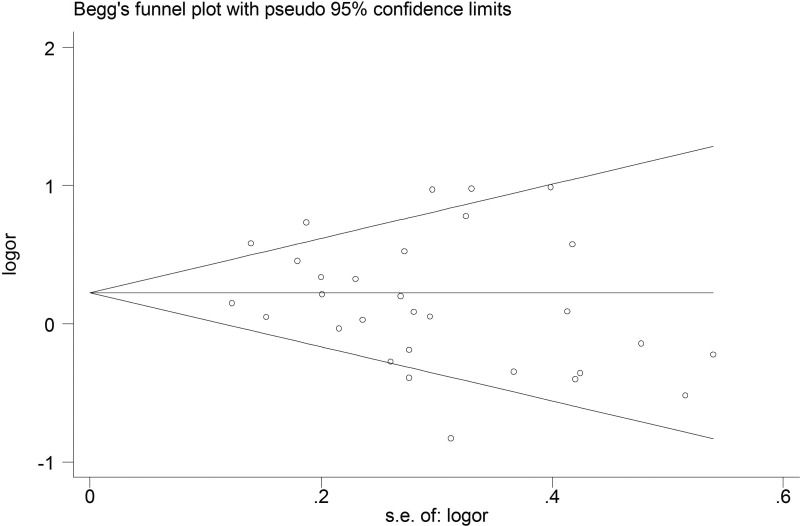
Begg's funnel plot analysis of GSTT1 polymorphism with esophageal cancer risk

Publication bias was assessed by both the Begg’s funnel plot and the Egger’s test. The shape of the Begg’s funnel plot did not reveal any evidence of obvious asymmetry (Figure 5). Egger’s test further suggested no evidence of publication bias (*P* = 0.210). Thus, there was no obvious publication bias in this meta-analysis.

**Figure 6:**
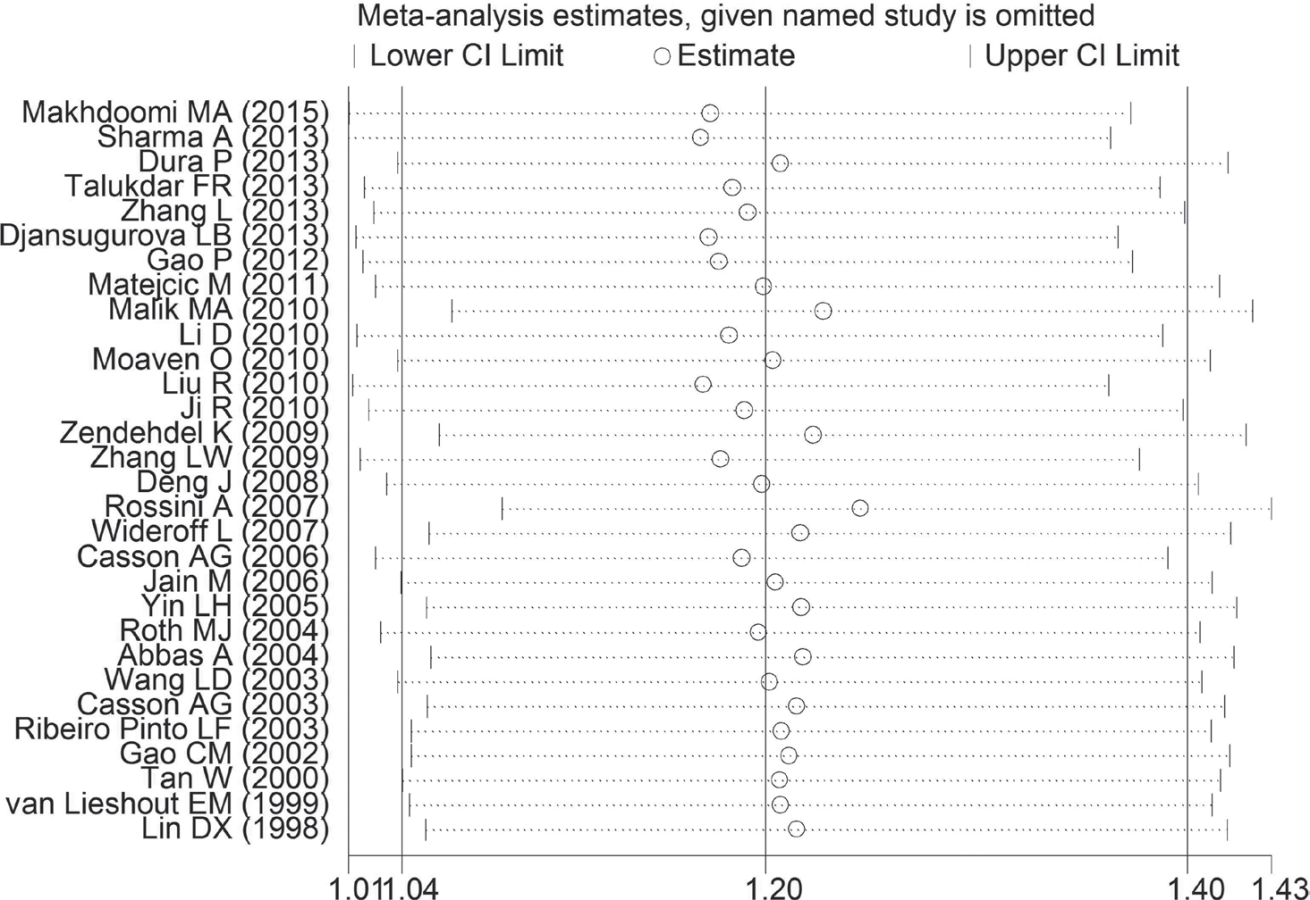
One-way sensitivity analysis of GSTT1 polymorphism with esophageal cancer risk

The sensitivity analysis was conducted to test the influence of an individual data on the pooled ORs and CIs by eliding a study in turn. Our findings suggested that the present meta-analysis results were relatively robust and stable (Figure 6).

As showed in Figure 2 and Figure 3, heterogeneity was significant in overall and in some subgroups. Thus, we measured the sources of heterogeneity by subgroup analysis. The results showed that Caucasians may lead to the major source of heterogeneity.

## DISCUSSION

Many studies suggest genetic variants play important roles in individual susceptibility to esophageal cancer [3, 42]. In decades, epidemiological studies have been performed to assess the association of GSTT1 null genotype with the risk of esophageal cancer. However, the results were inconsistent [34, 36]. Previous meta-analyses also investigated the association [12–14], the findings should be interpreted with very cautions. In Weng *et al.* study, 11 studies included in the meta-analysis were in Chinese Han population. Although the results showed a significant association between GSTT1 null genotype and esophageal cancer risk, the single Chinese Han population limited the power of the statistical analysis [12]. Also In Yi *et al.* study, 15 studies included in the meta-analysis were in Asian population. Although the results showed a significant association between GSTT1 null genotype and esophageal cancer risk, the single Asian population limited the power of the statistical analysis [13]. In Cai *et al.* study, a total of 24 studies were used. Adjusted ORs with corresponding 95% CIs were reported in 9 studies. In the overall analysis there was no significant association between GSTT1 null genotype and esophageal cancer risk. However, meta-analysis of adjusted ORs showed a significant association between GSTT1 null genotype and esophageal cancer risk [14]. Because of the lack of available data, subgroup analysis by histological type was not performed in these studies [12–14]. Thus we conducted a comprehensive meta-analysis, to investigate not only the strength of association between GSTT1 null genotype and the risk of esophageal cancer, but also the association of GSTT1 null genotype with the risk of different histological types.

This meta-analysis, including 30 case-control studies with 4482 cases and 6681 controls, identified the association between GSTT1 null genotype and esophageal cancer risk. GSTT1 null genotype significantly increased overall esophageal cancer risk. In a subgroup analysis by ethnicity, GSTT1 null genotype was correlated with a significantly increased risk of esophageal cancer among Asians. In the analysis by histological type, GSTT1 null genotype was correlated with a significantly increased risk of ESCC, particularly in Asians. However, there was no significant correlation of GSTT1 null genotype with the risk of EADC. To date, this is the first meta-analysis concerning the association of GSTT1 null genotype with the risk of different histological types.

GSTT1, encodes an enzyme that plays a crucial role in the detoxification of a variety of endogenous or exogenous carcinogens. It is located on 22q11.23 with 8146 base pairs, 5 exons and 4 introns in all [5]. GSTT1 is genetically polymorphic, and GSTT1 null genotype results in the loss of functional activity [12]. Our results demonstrated that GSTT1 null genotype significantly increased overall esophageal cancer risk.

Since the results from meta-analysis can be affected by histological types, a subgroup analysis was carried out regarding different histological type for the GSTT1 null genotype. GSTT1 null genotype was correlated with a significantly increased risk of ESCC among Asians, but not among Caucasians and Africans. However, there was no significant correlation of GSTT1 null genotype with the risk of EADC. All results should be interpreted with caution. Only two African studies were recruited in the current meta-analysis, which may restrict statistical power to detect a real assessment in Africans. More large scale studies are needed to verify the results. Subgroup analyses were also performed regarding ethnicity for the GSTT1 null genotype. GSTT1 null genotype was correlated with a significantly increased risk of esophageal cancer among Asians, but not among Caucasians and Africans. The results were more robust on histological type of ESCC. This meta-analysis confirmed the mutual effect of GSTT1 null genotype in different populations to the risk of esophageal cancer. Possible explanations include: (1) significance of these enzymes may vary with the ethnicity genetic backgrounds, environmental exposures and histological types. The regional difference in the frequency of esophageal cancer is probably due to genetic polymorphism and variable exposure to environmental factors; (2) GSTs metabolize a variety of overlapping substrates and individuals lacking GSTT1 can also metabolize the carcinogens by other alternative GST enzymes. Furthermore, there was only one study concerning the association between GSTT1 null genotype and EADC on Asians. Thus, we failed to evaluate the potential role of GSTT1 null genotype in EADC risk in Asians due to the lack of available data to date. More case-control studies on the GSTT1 null genotype are encouraged, especially in Asians, for a better understanding the role of GSTT1 null genotype in the EADC development.

Some limitations must be acknowledged in the current meta-analysis. First, significant heterogeneity was observed between publications for GSTT1 null genotype. Potential sources of heterogeneity include the study design, publication year, ethnicity, country, histological type, sample size, and so on. When subgroup analyses were carried out according to ethnicity and histological type, this heterogeneity was reduced or removed in some subgroups, implying different effects on histological types and ethnic populations. These findings should be interpreted with very cautions. Second, our findings were based on unadjusted ORs and CIs, whereas a more precisely investigation could be performed if the sufficient individual data were available. Third, only two African studies were recruited in the current meta-analysis, the results in African population should be interpreted with caution. More large scale studies are needed to verify the results in Africans. Finally, due to lack of uniform individual-level data, further stratified analysis to measure any interactions between gene–gene variation and gene-metabolic traits was not performed.

In conclusion, our meta-analysis findings demonstrated that GSTT1 null genotype significantly increased esophageal cancer risk, particularly in Asians. In addition, GSTT1 null genotype was correlated with a significantly increased risk of ESCC, particularly among Asians. However, more studies are warranted to confirm or refute these correlations, particularly with respect to gene-gene and gene-environment interactions.

## MATERIALS AND METHODS

### Study selection

Pubmed, Embase, China National Knowledge Infrastructure (CNKI) and Wanfang databases (the search was updated in March 31, 2017) were searched using the following terms: ‘glutathione S-transferase T1’ or ‘GSTT1’, ‘polymorphism’ or ‘variant’, and ‘esophageal’ or ‘esophagus’, and ‘cancer’ or ‘carcinoma’ or ‘tumor’ or ‘malignancy’. The literature search was limited to English or Chinese articles. Additional publications were identified by a manual search based on references of retrieved studied or reviews.

### Inclusion and exclusion criteria

The selection criteria were: (1) in a case-control study design, (2) studies that evaluated the relationship between the GSTT1 null genotype polymorphism and esophageal cancer, (3) usable data on genotype frequency. Thus, reports without usable data, reviews, comments and duplicated publications were excluded.

### Data extraction

The data were collected by two independent reviewers. The extracted information contained: first author, year of publication, country of origin, ethnicity, number of cases and controls, genotyping method and characteristics of cases and controls. When come to conflicting assessment, disagreements were settled through a discussion among all authors.

### Statistical analysis

The strength of correlation between GSTT1 null genotype and the susceptibility of esophageal cancer was assessed by the crude odds ratios (ORs) with 95% confidence intervals (CIs). A *P* < 0.05 (two-tailed) was considered as statistical significance. A Chi-square-based *I*^*2*^ test was used to detect heterogeneity [43] and an *I*^*2*^ < 25% indicates low heterogeneity, 25% ≤ *I*^*2*^ ≤ 50% indicates moderate heterogeneity, and *I*^*2*^ > 50% indicates large heterogeneity [44]. When *I*^*2*^ > 50% or *P* < 0.10 (two-sided), the random-effects model (the DerSimonian-Laird method) was utilized to pool the data [45], otherwise the fixed-effects model (the Mantel-Haenszel method) was used [46]. Subgourp analyses were conducted according to different ethnicity to identify the specific effects of heterogeneity. Publication bias was assessed by Begg’s funnel plot and Egger’s test [47]. Sensitivity analyses were conducted by one-way method. All statistical analyses were performed using STATA version 12.0 software (Stata Corporation, College Station, TX, USA).

## Abbreviations

Glutathione-S-Transferase T1: GSTT1
China National Knowledge Infrastructure: CNKI
odds ratios: ORs
confidence intervals: CIs
esophageal squamous cell carcinoma: ESCC
esophageal adenocarcinoma: EADC
